# Oral Dalfampridine Improves Standing Balance Detected at Static Posturography in Multiple Sclerosis

**DOI:** 10.1155/2014/802307

**Published:** 2014-03-27

**Authors:** Luca Prosperini, Costanza Giannì, Deborah Fortuna, Maria Rita Marchetti, Carlo Pozzilli

**Affiliations:** ^1^MS Centre, Department of Neurology and Psychiatry, Sapienza University, Viale dell'Università, 30-00185 Rome, Italy; ^2^Physical Therapy Unit, S. Andrea Hospital, via di Grottarossa, 1035-00189 Rome, Italy

## Abstract

We report a 14-week post-marketing experience on 20 patients with multiple sclerosis (MS) who started prolonged-release (PR) oral dalfampridine 10 mg twice daily according to European Medicine Agency criteria. They underwent serial static posturography assessments and the dizziness handicap inventory (DHI) to investigate whether PR dalfampridine could impact standing balance and self-reported perception of balance. The incidence of accidental falls per person per month was also recorded throughout the study. Eight (40%) patients, who had a relevant improvement in walking speed, were defined as treatment responders. They showed a significant improvement of standing balance (with respect to pretreatment assessment) when contrasted with 12 (60%) nonresponders (*F*_[4,15]_ = 3.959, *P* = 0.027). No significant changes in DHI score, as well as in its functional, physical, and emotional subscales, were found in both responders and nonresponders at the end of study (all *P* values are ≥0.2). Treatment response did not affect the incidence of accidental falls. Future studies based on larger sample sizes, and with longer followup, are required to confirm the beneficial effect of PR dalfampridine on standing balance.

## 1. Introduction

Reduced mobility, especially in walking, is probably the impairment most frequently accounted as compromising daily life activities of patients with multiple sclerosis (MS) [1]. Lack of balance/coordination is reported by patients with MS as one of the most common symptoms affecting mobility (67%), together with leg weakness (81%), fatigue (73%), and inability to walk long distances (69%) [2].

Lack of balance in MS remains largely incurable [3], since pharmacological interventions to improve balance are often inadequate, and some drugs broadly used in neurological setting may even worsen gait and balance [4].

Prolonged-release (PR) oral dalfampridine, a voltage-gated potassium channel blocker able to improve action potential conduction in demyelinated axons, is the first drug specifically licensed to improve walking in patients with MS [5]. Several randomized clinical trials demonstrated that PR dalfampridine can improve walking speed by approximately 25% in more than one-third of treated patients; moreover, it can significantly improve perception of ambulatory performance and lower extremity strength [6–9]. However, the potential effectiveness of PR dalfampridine in ameliorating balance is still debated, and an international, multicenter clinical trial is ongoing to address this important issue (NCT01597297) [10].

On the other hand, higher incidence of dizziness and balance disorders has been described as a common adverse event in PR dalfampridine-treated patients [8, 9].

In this study, we investigated whether PR dalfampridine could impact standing balance, perception of balance, and incidence of accidental falls in a cohort of patients with MS.

## 2. Methods

### 2.1. Participants

A total of 20 consecutive patients with MS according to McDonald criteria [11], who started commercial available PR dalfampridine tablets (10 mg per os, twice daily) according to European Medicine Agency criteria [12], were recruited at MS Centre of S. Andrea Hospital, Sapienza University, Rome, Italy. Eligibility criteria were age from 18 to 65 years; Expanded Disability Status Scale (EDSS) [13] score from 4.0 to 7.0 (inclusive); ability to complete two trials of the 25-foot walking test (25-FWT) [14] in a maximum time of 180 seconds; ability to stand upright for at least 180 seconds without any support. We excluded patients with history of seizures, concomitant otological or vestibular diseases (non-MS related), psychiatric comorbidities or severe cognitive impairment, and cardiovascular and respiratory disorders. An informed consent was obtained for each patient before any study procedure. The local ethical committee board provides exemption of approval for postapproval prospective studies.

### 2.2. Study Design

This was a 14-week, independent, single-centre, observational, postmarketing study.

Patients entered into the study at WK−4 visit. During the initial 4-week screening period they neither did receive PR dalfampridine nor changed their concomitant therapies. Administration of PR dalfampridine started for all patients at WK 0 visit, and response to treatment was assessed after a 2-week run-in period, according to previously established criteria based on 25-FWT [7]. A patient was defined as a responder if he/she walked faster in 3 out of 4 trials performed at the end of 2-week run-in period (WK+2 visit) versus the fastest performance of 5 weekly screening evaluations. Treatment responders continued the treatment with PR dalfampridine, while nonresponders discontinued it. However, we continued to evaluate both groups every 4 weeks (WK+6 and WK+10 visits).

**Figure 1:**
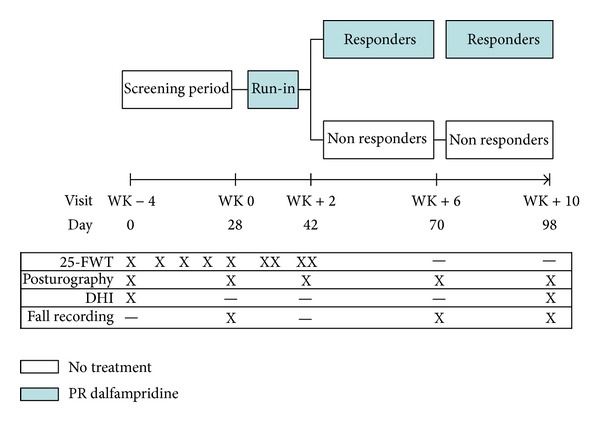
Study design and assessments. PR: prolonged release; 25-FWT: 25-timed foot walking test; DHI: dizziness handicap inventory.

A +/− 3-day window was allowed for each visit, with exception of WK 0 visit (when treatment response had to be defined).  Figure 1 shows the study design.

### 2.3. Study Assessments

*Walking Speed*. The 25-FWT was performed at study entry (WK−4) and every week up to WK 0 visit, for a total of 5 evaluations (screening period). Two 25-FWT trials, separated by a 30-minute interval, were also performed at the end of the first week and the second week of treatment (run-in period), for a total of 4 evaluations.

*Static Posturography*. Patients were tested by means of a laboratory-grade force platform (ProKin, Tecnobody, Dalmine, Italy; 20 Hz sampling rate, sensitivity 0.1°) at each visit by two trained physical therapists (DF and MRM), unaware of clinical data and treatment response status. Static posturography was performed according to a standardized procedure as follows: each subject was asked to stand barefoot on the ground, in upright static condition, double-leg stance and with arms resting at their sides. The position of the feet on the force-platform was standardized using a V-shaped frame, keeping on a 3 cm distance between the two medial malleoli and an extra rotation of 12° with respect to the sagittal axis [15]. The sum of displacement of body's centre of pressure (COP) under open-eye condition in 30 seconds was measured to estimate the extent of postural sway; wider sway indicates worse standing balance. This parameter has been reported to be highly reliable (95%) and also more accurate than clinical scales in predicting accidental falls [16].

*DHI*. The questionnaire was administered to patients at study entry (WK−4) and study termination (WK+10). The DHI is a 25-item self-administered questionnaire rating the self-perception of disability due to dizziness and imbalance [17]. The total score ranges from 0 to 100, with higher score indicating more severe disability. Subscores related to physical, emotional, and functional domains can be also calculated. DHI exhibited an excellent test-retest reliability (90%), and a 50% sensitivity and 74% specificity in detecting fallers among patients with MS were also reported when a cut-off score of 59 was used [18].

*Accidental Falls*. At study entry (WK−4), and at WK+2 and WK+6 visits, all patients received a diary to fill out and give back the next visit, in order to estimate the month incidence of accidental falls (i.e., an unexpected contact of any part of the body on the ground).

### 2.4. Statistical Analysis

Baseline differences between responders and nonresponders were tested by the Mann-Whitney* U* or the Fisher Exact tests for continuous and categorical variables, respectively.

A repeated measures analysis of variance (ANOVA) was carried out to estimate the main effects of time (within-subject factor) and time by group (responders versus nonresponders, between-subject factor), with log-normalized COP path values at different time-points as dependent variable. Data on COP path were log-transformed since they were not normally distributed. Mauchly's test indicated that the sphericity assumption was satisfied; therefore correction for degrees of freedom was not necessary.

Changes over time in DHI and its subscales were investigated by the Wilcoxon Rank Sum test.

The incidence rate ratio of falls per person per month was also investigated for responders and nonresponders.

*P* values less than 0.05 in either direction were considered as statistically significant. Analyses were carried out by using the Statistical Package for Social Sciences, version 16.0 (IBM SPSS, Chicago, IL, USA).

## 3. Results

Demographic and clinical characteristics of study sample at study entry are shown in Table 1. After the 2-week run-in period, 8 (40%) patients resulted as responders and 12 (60%) as nonresponders. The mean walking speed improvement, assessed with 25-FWT, was 33% and 8% in responders and nonresponders, respectively (*P* = 0.001). No adverse events, other than accidental falls, occurred during the 14-week study period. None of the reported falls resulted in a major injury or fracture. No seizures were reported.

We did not find any significant main effect of time on postural sway (*F*_[4,15]_ = 1.591, *P* = 0.231), while a significant time by group interaction was observed (*F*_[4,15]_ = 3.959, *P* = 0.027), indicating a reduced postural sway in the treatment responder group.

**Figure 2:**
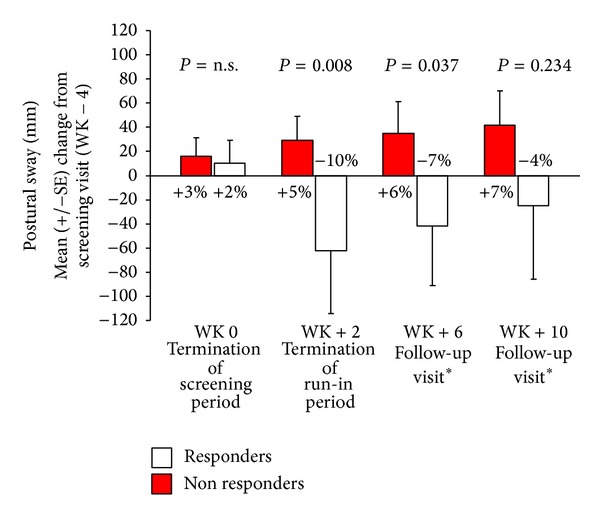
Mean absolute difference (±standard error, SE) in COP sway displacement (mm) from screening (WK−2) value. Negative values refer to improved standing balance. SE: standard error. *PR dalfampridine taken only by treatment responders.

Simple contrast analyses revealed a significant improvement in postural sway of responders at WK+2 (*F*_[1,18]_ = 9.346, *P* = 0.008; mean change from visit WK−4: −10%) and WK+6 (*F*_[1,18]_ = 5.193, *P* = 0.037; mean change from visit WK−4: −7%), but not at the end of study (*F*_[1,18]_ = 2.855, *P* = 0.234; mean change from visit WK−4: −4%) compared to pretreatment values. Postural sway of nonresponders tended to worsen, with a 7% increase of postural sway at study termination (see Figure 2).

There were no significant changes from WK−4 to WK+10 visits in DHI total score neither in treatment responders (*P* = 0.121) nor in nonresponders (*P* = 0.482). Similar findings were found even considering DHI subscales (functional, physical, and emotional), with nonsignificant *P* values from 0.293 to 0.150 and from 0.959 to 0.191 in treatment responders and nonresponders, respectively.

Similar rates of accidental falls per person per month were observed in treatment responders (0.875 from WK−4 to WK 0 visits; 0.625 from WK+2 to WK+6 visits; 0.375 from WK+6 to WK+10 visits) and nonresponders (0.833 from WK–4 to WK 0 visits; 0.667 from WK+2 to WK+6 visits; 0.375 from WK+6 to WK+10 visits) (*P* = 0.82).

## 4. Discussion

The main finding of this study is that response to PR dalfampridine was associated with significant, short-term, improvement in standing balance (detected at static posturography).

Moreover, there was a relevant and sustained dissociation between responders and nonresponders in terms of postural sway differences with respect to pretreatment performance, even though treatment responders returned toward baseline values. These latter findings might be explained by the progressive nature of the disease. However, we cannot exclude the hypothesis of a “boost” effect of PR dalfampridine, which tends to decrease over time. Consistently with this hypothesis, a recent postmarketing experience has reported significant improvement in walking speed only after two weeks from treatment start, not confirmed on a longer-term period [19]. Conversely, the beneficial effect of PR dalfampridine on other clinical parameters, such as maximum walking distance, and patient-reported outcome of motor and cognitive fatigue, was maintained up to 12 months from treatment start [19].

The significant improvement in standing balance of treatment responders might be explained by at least three distinct (and perhaps mutually reinforcing) mechanisms.

First, by quickly restoring nerve conduction, probably driven by an enhanced excitatory synaptic transmission [5, 20], PR dalfampridine might be able to improve the integration of afferent signals into the central nervous system. Central integration deficit, due to the widespread and variable distribution of demyelination and axonal loss, can be considered as a major contributor of balance deficit in MS [21].

Second, PR dalfampridine might improve anticipatory postural adjustments by increasing lower extremity strength. Lesser directional-specific and smaller magnitudes of anticipatory muscle activation were in fact reported even in mildly disabled patients with MS [22].

Third, PR dalfampridine might enhance the inhibitory drive and precision of pacemaking of cerebellar Purkinje cells, as also supported by animal studies on its analogue 4-aminopyridine (4-AP) [23]. Some studies showed the ability of 4-AP in ameliorating not only specific cerebellar dysfunctions, such as type-2 episodic ataxia and downbeat nystagmus [24], but also upper limb tremor and dysarthria due to MS [25, 26].

The role of cerebellum damage in determining standing balance impairment has been also recently emphasized in nonconventional magnetic resonance imaging studies [27], thus reinforcing this latter hypothesis.

Among the PR dalfampridine-related adverse events, randomized clinical trials described an increased incidence of dizziness and balance disorders, especially in the first 4–8 weeks of treatment. Nevertheless, a similar incidence of accidental falls in active and placebo arms [8, 9] has been reported. Consistently with clinical trials, the incidence of fallers was similar in responder and nonresponder group in our experience, suggesting that response to PR dalfampridine could not be considered a risk factor for accidental falls. Moreover, patients' self-perception of disability due to balance problems, as assessed by DHI and its subscales, was not affected by treatment response to PR dalfampridine. However, the interpretation of these latter findings, especially that one about the risk of accidental falls, must be cautious, given the small population enrolled in our study.

The major limits of our study mainly encompass its observational, nonrandomized design, the lack of a placebo-controlled group, and the small sample size. However, the use of computer-based measures of standing balance, that are accurate and reliable in MS setting [28], provided data more objective than clinical scales.

Findings from our study require to be confirmed by further prospective studies on larger sample sizes and even on a long-term period.

## Figures and Tables

**Table 1:** Patients' characteristics at study entry.

	Responders *n* = 8	Nonresponders *n* = 12	Pooled *n* = 20
Gender: F : M	4 : 4	6 : 6	8 : 12
Age: years	45.2 (8.1)	44.6 (6.4)	44.8 (7.1)
MS duration: years	14.5 (7.9)	14.7 (6.9)	14.7 (7.2)
Disease course: RR : SP : PP	2 : 5 : 1	4 : 6 : 2	6 : 11 : 3
EDSS score: median [range]	5.0 [4.0–6.5]	5.0 [4.0–7.0]	5.0 [4.0–7.0]
Walking speed: m/s	0.71 (0.34)	0.87 (0.44)	0.81 (0.42)
COP sway path: mm	625 (317)	580 (357)	599 (333)
DHI score	44.0 (15.1)	47.8 (22.1)	46.2 (17.7)

All values are presented as mean (standard deviation), unless indicated otherwise.

All P values are >0.2.
